# “If I Start [Primary Prevention] Now, I Can Prevent [Cancer]”: College Students Endorse Cancer Prevention Education

**DOI:** 10.1007/s13187-025-02574-6

**Published:** 2025-02-19

**Authors:** Jacqueline Knight Wilt, Maria D. Thomson

**Affiliations:** Department of Social and Behavioral Sciences, School of Public Health, Virginia Commonwealth University, One Capital Square, Box 980430, Richmond, VA USA

**Keywords:** Colorectal cancer, Early onset, Perceived risk

## Abstract

Early onset colorectal cancer has been linked to lifestyle-related risk factors. Emerging adulthood (ages 18–25) has the greatest changes to lifestyle health behaviors with implications for health outcomes. College students have moderate consideration of future cancer risk (CFC-CA) when navigating current health behavior choices. This study explored cancer prevention knowledge, attitudes and behavioral intentions in a subset of students with low, medium and high CFC-CA. Qualitative interviews were used to explore cancer prevention among a sample of college students. We identified a stratified sample (*N* = 43) of high CFC-CA (*n* = 16), medium CFC-CA (*n* = 14), and low CFC-CA (*n* = 13) students who agreed to complete a 60-min interview on Zoom. Interviews were transcribed verbatim, and transcripts were coded using an iterative, line-by-line approach. Group comparisons were completed after coding was complete. Students exhibited greatest awareness of highly publicized cancers including the lung, breast, and skin. Fifty-four percent of those with low CFC-CA and 87% with high CFC-CA believe that their current health behavioral intentions are protective against cancer. Most students want more cancer education regardless of CFC-CA level to know how they can reduce their cancer risk. Differences in preferred information sources (indirect vs direct) were identified for low versus higher CFC-CA, respectively. Students’ preferences for cancer education were related to their CFC-CA scores. This could be used to tailor information content and delivery modality. Delivering cancer prevention education through means that facilitate internal reflection may be more effective for improving risk reduction behaviors.

## Background

Cancer diagnoses and death among individuals too young for preventive population screening guidelines, recognized as early onset cancer, continue to rise globally [1]. In the USA, colorectal cancer among those age ≤ 50 is the second most common cancer diagnosis [2]. By 2030, projections from 1975 to 2010 estimate a 90% increase of colon and 124% of rectal cancers among individuals aged 20 to 34 years [3]. Early onset colorectal cancer (EOCRC), defined as colorectal cancer occurring before age 50 [4], has been linked to lifestyle-based risk factors including, obesity, diet, physical activity, alcohol use, smoking tobacco, and stress [5, 6].

Emerging adulthood (ages 18–25) encompasses some of the most substantial changes to health behaviors that are sustained into adulthood and affect the development of chronic illnesses including cancer [7]. In an Australian cohort, increasing physical activity during emerging adulthood was associated with healthier dietary patterns, better sleep, and overall self-rated health at ages 60–64 [8]. Another study found that 39% of emerging adults with protective behavior patterns sustained behaviors in young adulthood (26–31 years), 25% adopted more protective behaviors, and only 12% higher risk behaviors [7]. Given that health behaviors established during emerging adulthood may have a sizable impact on EOCRC risk, the extent to which emerging adults are thinking about near and longer-term cancer risk is of increasing importance. Being more future-goal oriented when making behavioral decisions, measured as consideration of future consequences, is linked to health promoting lifestyle behaviors among college students [9]. We adapted this scale to measure consideration of future cancer risk (CFC-CA) when navigating health behavior choices and found that college students’ decisions are moderately motivated by reducing cancer risk [10]. Higher CFC-CA was associated with more health promoting behaviors for diet, sedentariness, alcohol use, smoking, and stress management [10].

Behavioral intentions are a predictor of health behavior and are influenced by psychosocial variables including attitudes, norms and self-efficacy [11, 12]. Sun protective behavior among college students is facilitated by intentions to limit sun exposure, self-efficacy in sunscreen use, and beliefs in the importance of these behaviors [12]. An intervention study found that while increasing positive attitudes and beliefs about physical activity increased intentions, academic requirements and work schedules remained significant barriers [13]. These barriers reflect skills and environmental constructs of the Integrative Model of Behavioral Prediction (IMBP) [14]. IMBP identifies health behavior as directly related to behavioral intention and moderated by skills and environmental factors. A systematic review of IMBP-informed studies identified that very few assessed skills and environmental factors [15]. This is a significant gap in our understanding of student behaviors given the changes in environment and autonomy experienced by college students.

Projected EOCRC trends signals need for lifestyle-focused primary prevention approaches that target individuals earlier in the life course. Greater CFC-CA is associated with more health promoting lifestyle behaviors [10]; thus, there may be differences in behavioral intentions or psychosocial factors across CFC-CA levels. The purpose of this study was to explore knowledge, attitudes, and cancer prevention behavior intentions among college students across the spectrum of low, medium, and high CFC-CA scores. Investigating potential differences across CFC-CA groups can identify modifiable factors for future interventions to increase EOCRC primary prevention behaviors.

## Materials and Methods

### Data Sample

A stratified sample of college students ages 18–25 years at a 4-year public institution in the USA who completed a web-based survey was recruited for follow-up interviews. Survey measures have been described in a prior manuscript [10]. Briefly, they included a cancer-specific adapted version of the consideration of future consequences scale to assess CFC-CA, perceived colorectal cancer risk, stress, colorectal cancer knowledge, and sociodemographic characteristics.

Students were stratified into three groups based upon CFC-CA score categorized as low (score range 21–54), medium (55–67), and high CFC-CA (68–95). The sample’s mean CFC-CA score (μ = 61) and one standard deviation around the mean (SD = 14) were used to delineate groups. Within each group, participants were randomly sorted, and the first 20 for each group were invited to participate. Email invitations included a link to schedule an interview if they chose to participate. In total, 125 students across low (*n* = 39), medium (*n* = 49), and high (*n* = 37) were invited, and final sample included 13 low, 16 medium, and 14 high CFC-CA participants.

Interviews were conducted using the institutional, secure Zoom. Verbal consent to participate was conducted prior to the interviews. Interviews lasted approximately 60 min, and recordings were transcribed verbatim. A $20 gift card compensated participants for their time. Study protocol was reviewed and determined exempt by the university’s Institutional Review Board (HM20026461).

### Interview Guide

The semi-structured interview guide was developed with input from undergraduate students. Open-ended questions guided by the integrative model of behavioral prediction [14] asked about cancer prevention knowledge, attitudes, and risk perceptions before inquiring about their health behavior intentions. Participants were asked about health behavioral intentions as a student, and 5 years in the future. Students then reflected on how these relate to their future cancer risk. Participants were asked to describe barriers and facilitators for enacting behavioral intentions and to provide recommendations for campus resources to support health promoting behaviors.

### Analysis

Interview participants’ survey responses were pulled for descriptive and comparative statical analyses in SPSS 28 [16]. Interviews were transcribed verbatim and uploaded into MAXQDA 2024 [17]. Principles of the Grounded Theory approach were utilized to analyze transcripts individually and then compare across groups [18]. A set of transcripts were initially open coded to develop a codebook which was iteratively revised over the course of the project. The research team reviewed final set of codes and interview notes to make observation of emergent themes. MAXQDA’s compare groups analysis feature was used to examine the frequency of themes across groups.

## Results

### Participants

Characteristics of the full sample and by group are displayed in Table 1. Mean age was 20 years (SD = 1.5) and the majority identified as female (81%). A quarter were first year students, and 40% were fourth or fifth year. Around a third (30%) received need-based financial assistance, and 20% were first generation college students. The sample reported moderate stress (mean = 20.5), less than half lived in campus housing, half worked at least part-time while in school, and about half (56%) had family history of cancer. Mean CRC knowledge score was 14.5, on scale of 0–18, and majority believed their likelihood of getting CRC in the future was low or very low. Only GPA was significantly different across the three groups, F(2) = 3.792, *p* = 0.03.

**Table 1:** Full interview sample and by group characteristics

	Full sample	Low CFC-CA	Mean CFC-CA	High CFC-CA
	*N* = 43	*N* = 13	*N* = 16	*N* = 14
Age	20 (SD = 1.5)	20 (SD = 1.5)	20 (SD = 1.4)	19 (SD = 1.4)
Sex				
Male	18.6% (8)	15.4% (2)	12.5% (2)	28.6% (4)
Female	81.4% (35)	84.6% (11)	87.5% (14)	71.4% (10)
Race/ethnicity				
Asian	34.9% (15)	30.8% (4)	43.8% (7)	28.6% (4)
Black	11.6% (5)	7.7% (1)	12.5% (2)	14.3% (2)
Latino	4.7% (2)	7.7% (1)	0%	7.1% (1)
White	41.9% (18)	53.8% (7)	31.3% (5)	42.9% (6)
Bi/multiracial	4.7% (2)	0%	6.3% (1)	7.1% (1)
Other	2.3% (1)	0%	6.3% (1)	0%
Year in school				
First year	25.6% (11)	23.1% (3)	18.8% (3)	35.7% (5)
Second year	14.0% (6)	15.4% (2)	12.5% (2)	14.3% (2)
Third year	20.9% (9)	15.4% (2)	25.0% (4)	21.4% (3)
Fourth year +	39.6% (14)	46.2% (6)	43.8% (7)	28.6% (4)
GPA*	3.5 (SD = 0.7)	3.1 (SD = 0.6)	3.6 (SD = 0.6)	3.7 (SD = 0.6)
Program of study				
Arts	11.6% (5)	7.7% (1)	12.5% (2)	14.3% (2)
Business	7.0% (3)	7.7% (1)	12.5% (2)	0%
Engineering	4.7% (2)	0%	6.3% (1)	7.1% (1)
Government	9.3% (4)	23.1% (3)	0%	7.1% (1)
Health professions	11.6% (5)	15.4% (2)	6.3% (1)	14.3% (2)
Humanities	58.1% (25)	53.8% (7)	56.3% (9)	64.3% (9)
Life sciences	7.0% (3)	7.7% (1)	6.3% (1)	7.1% (1)
Nursing	4.7% (2)	7.7% (1)	6.3% (1)	0%
Double major	14% (6)	23.1% (3)	6.3% (1)	14.3% (2)
First Generation college student	20.9% (9)	30.8% (4)	25.0% (4)	7.1% (1)
Need-based financial aid recipient	30.2% (13)	46.2% (6)	31.3% (5)	14.3% (2)
Employed ≥ part-time while in school	51.2% (22)	61.5% (8)	50.0% (8)	42.9% (6)
Live in campus housing	39.5% (17)	53.8% (7)	31.3% (5)	35.7% (5)
Current perceived stress	20.5 (SD = 5.7)	22.6 (SD = 6.1)	20.4 (SD = 5.5)	18.8 (SD = 5.3)
Family history of cancer	55.8% (24)	61.5% (8)	43.8% (7)	64.3% (9)
CRC knowledge	14.5 (SD = 2.4)	14.9 (SD = 1.3)	13.7 (SD = 3.0)	15.2 (SD = 2)
Perceived likelihood of CRC in future				
Very low	32.6% (14)	46.2% (6)	31.3% (5)	21.4% (3)
Low	41.9% (18)	38.5% (5)	43.8% (7)	42.9% (6)
Moderate	20.9% (9)	7.7% (1)	18.8% (3)	35.7% (5)
High	2.3% (1)	7.7% (1)	0%	0%

^*^p value ≤ .05

### Themes

Four themes were identified: existing cancer knowledge, integrating cancer preventive action during college, cancer prevention will be easier in the future, and cancer prevention education endorsement. Excerpts from student interviews are displayed in Table 2.

**Table 2:** Emerging theme descriptions and student excerpts

Existing Cancer Knowledge Theme: Exhibiting existing knowledge of cancer prevention and control		
* Risk factors subtheme: Exhibiting knowledge of genetic, familial, environmental or lifestyle-related cancer risk factors*	Low CFC-CA	“I know like avoiding cancerous products. Like there’s chemicals in some of the like the foods that we eat that like are like, Oh, warning this and that. I know like they always have warnings selling cigarettes, some hair products like hairspray and stuff like that” -Female, age 18
	Medium CFC-CA	“And sometimes even genetics, if like my grandparents, both my grandmother's had cancer at some point in their life. So I think it could be genetic or it depends on like, old age or something” -Female, age 20
	High CFC-CA	“I know that heredity, heredity, heredity, so like family risk plays a role in that as well. And I also believe exposure, like exposure to certain types of chemicals, workplace chemicals, or even and I consider like smoking to be a type of exposure to you know, those types of harmful chemicals. So I feel like these kind of are the four main things. So family risk, diet, exercise. And then exposure” -Male, Age 20
* Prevention and detection subtheme: Exhibiting knowledge of cancer screenings, self-exams or immunotherapy*	Low CFC-CA	“So. All I know is that like, you can really should check your own your like your genitals to see if there's any lumps” -Male, age 21
	Medium CFC-CA	“Um, I think the only one that I'm like really aware of is the breast cancer screenings… and maybe skin cancer, I just, I don't really know, the screening precautions. I just know what to look for. Like, if you have a particular mole that's like, different color, it's not symmetrical and it just popped out of nowhere” -Female, age 21
	High CFC-CA	“I do know that you get them at a certain age, especially for women, you have to do the, like your Pap smears and your mammograms. And then for men, I think it's prostate exams. And then if in general, if you find like a weird lump, you go and get it checked out. Or if you have like a mole or anything, you get that checked out as well” -Female, age 18
Integrating Cancer Prevention Theme: Description of preventive cancer behaviors and attitudes as a college student		
* Current cancer prevention efforts subtheme: Mention of current health behaviors that align with cancer prevention recommendations*	Low CFC-CA	“I do avoid like any kind of smoke inhalation like I’ve always like I’ve been offered like a cigarette or like weed and I can’t, I won’t do that” -Female, age 21
	Medium CFC-CA	“I’m putting on sunscreen. I’m like sunscreen crazy fanatic…I always put on sunscreen, at least like in the morning… I'm definitely like, very strict about wearing sunscreen” -Female, age 21
	High CFC-CA	“I’ve been trying to use healthier products, like really good sunscreen, maybe deodorants that don’t have all these chemicals in it, as well as food, I tried to stay away from non-organic things. Of course, that’s hard for a student” -Female, age 18
* Future health status starts now subtheme: Exhibiting importance in developing health promoting practices now to shape future health*	Low CFC-CA	“Yeah, I think my intention overall is just to set myself up to be healthy as I get older. I don’t get to a point where I just feel burned out and tired” -Female, age 18
	Medium CFC-CA	“Like, it’s best to start early while you're young, to get these issues fixed out rather than when you are older. Because it just save you a long time, save you just overall in the long time when you are older” -Female, age 23
	High CFC-CA	“Well, I think it’s important because I want to stay healthy, and I want to live a long, long life. And I don’t want to get to a point where I’m old, and I’m dealing with many diseases and conditions. And if I start now, I can prevent that sort of regretting it later” -Female, age 18
* Prevention integration facilitators subtheme: Mention of factors that support carrying out current cancer prevention efforts*	Low CFC-CA	“Um, I really, truly think I just woke up and realized…I’m not going to be in high school again, where I’m not going to be in a position ever in my life where somebody is taking care of me, I have to take care of me. And so now I’m like, Yeah, nobody else is gonna do it. So I have to” -Female, age 21
	Medium CFC-CA	“And I’m also grateful that I have a good like, study schedule, and I know my habits pretty well. So I plan a lot time for these activities without having to do like, I don’t know whether I’ll have time to do this or not, like I know that I have time to put it into my schedule” -Female, age 18
	High CFC-CA	“I would say for in terms of easier, I would say other people that are also health conscious health minded. So the social peer aspect of it helps a lot” -Male, age 20
Cancer Prevention will be Easier after College Theme: Discussion of how carrying out cancer prevention behaviors will be easier five years in the future	Low CFC-CA	“Because I guess in five years, I’ll not be on my parents’ stuff [health insurance] so I definitely think that's something to think about in terms of like making sure I have a doctor or…at least like a doctor’s office like a primary care physician that I can like contact if I need help” -Female, age 20
	Medium CFC-CA	“I do think that like, certain things cost more money. So like I like I feel like to live a healthier lifestyle that it does cost a lot of money to do that” -Female, age 22
	High CFC-CA	“But like five years down the line, when like I have an established career…And you're just kind of like set, you're where you want to be in life. I think it’ll be easier to start focusing on yourself more” -Female, age 20
Cancer Prevention Education Endorsement Theme: Mention of desire to receive cancer prevention education earlier in the life course as an emerging adult	Low CFC-CA	“But there’s no general guidelines set in place that say…these are some preventative measures. Those are things you kind of have to go looking for and a lot of time people don’t do that type of research” -Female, age 20
	Medium CFC-CA	“But then there’s so many different types of cancers that you can get, but I feel like they’re not usually as readily talked about. So it's a put a little bit more publicity towards them and gave preventative factors that you could do for them. I think that could help quite a bit because it would inform students of just different daily habits that they could change in their lives” -Female, age 19
	High CFC-CA	“And like look more into like, what do I need to be doing [cancer prevention] that isn't like crystal clear. Like obviously, like smoke, don’t smoke, like that’s clear. Eat good. Try to exercise like those are like good things to do. But I want to see if I can find like, there's other things I need to be doing that could like help me” -Female, age 18

#### Existing Cancer Knowledge

When asked about current knowledge of cancer, two subthemes emerged across student interviews including risk factors and prevention and detection.

*Risk Factors – Students mentioned knowledge of genetic/familial risk for cancer most frequently in the high group (low 8%, medium 44%, high 57%). Environmental exposures to carcinogens, water and air contamination, and use of products like topical skincare, plastics, and cookware were commonly identified as risks. Several students across CFC-CA groups demonstrated knowledge of lifestyle-related health behaviors related to cancer risk. Behaviors mentioned mostly commonly included smoking and sun exposure (low 62%, medium 75%, high 71%), but also less commonly, diet, physical activity, and stress (low 39%, medium 50%, high 71%).*

*Prevention and Detection – Students discussed knowledge of screening guidelines for breast, skin, and gynecological cancers; colorectal was the least mentioned among students (16%). All participants in the high group mentioned cancer screening during their interviews (low 69%, medium 63%, high 100%); however, most did not know specifics about the types of screening tests available.*

#### Integrating Cancer Preventive Action During College

This theme represented student discussions around their current cancer prevention practices, health promoting behavioral intentions, and making time in one’s current schedule to engage in those activities.

#### Current Cancer Preventive Efforts

Majority of participants recounted engaging in cancer preventive behaviors related to skin, lung, breast, or gynecological cancers (low 77%, medium 69%, high 79%). Behaviors included regular sunscreen use, avoiding secondhand smoke, and regular gynecological exams. Most participants with high CFC-CA expressed a belief that their current behaviors contribute to lowering future cancer risk (low 54%, medium 56%, high 86%).

#### Future Health Starts Now

During the interviews, students expressed concerns about their future health and preventing health complications (low 69%, medium 56%, high 64%). Future health complications included cardiovascular disease, diabetes, chronic pain, and cancer. The idea of needing to start now to care for their future health was expressed. Some specifically mentioned needing to be proactive about cancer prevention in the present.

#### Prevention Integration Facilitators

Several factors supported preventive behaviors in the present. Half of participants across all groups mentioned health promoting preferences such as eating fresh fruits and vegetables and a dislike of alcohol. Students referenced having independence and their own living space as supporting their health behavior intentions. Scheduling time for health, like physical activity or food preparation, was mentioned in about half of participants. Familial and peer social support to perform preventive behaviors was endorsed among majority of the high group (low 54%, medium 38%, high 71%).

#### Cancer Prevention Will Be Easier After College

Students’ believed that cancer preventive action will be easier to implement five years from now. Due to competing priorities, personal health often fell on the “backburner.” The low CFC-CA group most often discussed having more finances, time, and comprehensive health insurance for prevention after graduation (low 77%, medium 31%, high 21%). Future intentions mostly included seeking out regular preventive healthcare, and a third want cancer screenings. Many discussed intentionality around sustaining or improving consistency for diet, physical activity, sleep, and stress management behaviors established during college.

#### Cancer Prevention Education Endorsement

Students mentioned wanting to know more about cancer and how to reduce risk (low 69%, medium 63%, high 86%). Half of low CFC-CA group remarked their study participation piqued interest in cancer prevention (low 54%, medium 13%, high 14%). Students listed cancer biology, risk factors, prevention strategies, and screening tests as items they want education on. Some mentioned a need to learn about the influence of current behaviors on future health and how to utilize health insurance to access preventive care (low 39%, medium 38%, high 14%). There were two opposing educational platforms which students recommended. Indirect means included print materials, emails, or webpage; direct means were courses, seminars, or individualized appointments. Indirect means were preferred among the low group, while medium and high preferred direct.

## Discussion

Students with high CFC-CA had greater knowledge of lifestyle-related risks for cancers. Participants across all groups mentioned currently using sunscreen and avoiding smoking as cancer prevention behaviors. Given highly visible campaigns like Truth Initiative to reduce teen smoking [19] and Pink Ribbon for breast cancer [20], it is not surprising that students were most aware of breast and lung cancer. Colorectal was only brought up in a handful of interviews even though it is the second leading cancer-related cause of death in the USA [21]. These findings suggest that breast and lung cancer public campaigns and education approaches could be replicated to bring awareness to EOCRC. Given EOCRC trends and the association with modifiable lifestyle health factors [5], it is encouraging that in this study, college students across the CFC-CA spectrum want knowledge about prevention strategies to reduce their own cancer risk.

Students expressed belief that future health begins to be shaped now and want to learn cancer prevention strategies they can implement. Current prevention efforts begin too late and focus on secondary prevention like age-based screening for early detection [22]. Primary prevention efforts should be prioritized to modify environmental and lifestyle-related EOCRC risk factors which have greater public health impact [23]. For instance, heavy alcohol consumption is a modifiable EOCRC risk factor and often occurs frequently among young adults for which the Centers for Disease Control and Prevention recommend screenings and brief interventions by healthcare providers [24]. This aligns with life course health development framework which suggests that younger age at initiation of preventive health behavior improves continuity [25]. Furthermore, this meets participants’ goals to sustain or improve consistency of protective lifestyle health behaviors established during college.

In 2021, cancer ranked among the top five leading causes of death, alongside unintentional injury and suicide, for those age 10–44 years in the USA [26]. The Substance Abuse and Mental Health Services Administration allocates funds to address unintentional injuries and suicide among college students [27]. While campuses nationwide address these immediate concerns for emerging adults, there is less focus on cancer prevention. Yet, there is increasing evidence that lifestyle plays a significant role in cancer risk and at younger ages [5]. Study participants expressed wanting to know more about cancer risk reduction, including lifestyle related risks. A comprehensive college health education curriculum that includes risk reduction strategies for diet, physical activity, substance use, mental health, and healthcare utilization could have extensive public health impact. Undergraduate students that engage in more leisure time physical activity have better mental health [28], and alumni that participated in required health courses have better health outcomes later in life [29]. Majority of participants didn’t know that HPV vaccination helps prevent cancer. In one study students recommended that colleges communicate vaccination benefits, potential risks, current guidelines, research of effectiveness, and availability at the campus health center and local clinics [30].

Several participants have annual physicals and plan to sustain or increase frequency to stay on top of their health. Annual physicals are opportunities to focus on primary prevention by assessing and advising lifestyle-related health behaviors to reduce cancer risk. A pilot study in the UK found that a brief lifestyle-related cancer risk assessment and intervention during annual appointments was feasible for providers and patients endorsed that the content was motivational for behavior change [31]. In the present study, high CFC-CA group preferred direct education like one-on-one appointments to learn cancer risk reduction strategies. Perhaps a brief intervention modeling the UK pilot could motivate activation of cancer prevention behaviors.

### Limitations

Participants self-selected to participate when invited to a follow-up interview which may reflect emerging adults more interested in cancer prevention compared with peers that chose not to participate. By stratifying the sample by CFC-CA, we ensured representation of students that are less future oriented regarding cancer prevention. Social desirability may have influenced responses about intentions for engaging in health-related behaviors. To encourage honest self-reflections the researcher opened interviews by expressing that the interest was about attitudes and experiences, thus, there was no such thing as a right or wrong answer. Over half Low CFC-CA group expressed interest in cancer prevention after study participation. This may reflect Hawthorne effect, in that research participation could unintentionally create behavior change [32]. However, this also justifies inclusion of emerging adults in future cancer prevention research as the exposure can increase awareness of lifestyle-related cancer prevention health behaviors.

## Conclusion

As evident from these student perspectives, emerging adulthood is not too young to begin discussing cancer risk reduction strategies, especially for lifestyle-related risk factors. Regardless of whether college students have high or low CFC-CA guiding their current health-related behaviors, they want to know more about cancer and how to reduce risk. Given cancer is among top 5 causes of mortality in this age group and rise of early onset cancers, we must intervene sooner that age-based screening recommendations. We are at a pivotal point to shift the cancer prevention paradigm to align primary prevention with an earlier life course framework for greater public health impact.

## Data Availability

The data that support the findings of this study are available from the corresponding author upon reasonable request.
